# Prevalence and Trends of Transfusion-Transmissible Viral Infections among Blood Donors in South of Iran: An Eleven-Year Retrospective Study

**DOI:** 10.1371/journal.pone.0157615

**Published:** 2016-06-16

**Authors:** Fatemeh Farshadpour, Reza Taherkhani, Saeed Tajbakhsh, Marziyeh Gholizadeh Tangestani, Gholamreza Hajiani, Nasrin Sharifi, Sakineh Taherkhani, Abdolreza Nejadbolkheyr

**Affiliations:** 1 Department of Microbiology and Parasitology, School of Medicine, Bushehr University of Medical Sciences, Bushehr, Iran; 2 Persian Gulf Tropical Medicine Research Center, Bushehr University of Medical Sciences, Bushehr, Iran; 3 Bushehr Blood Transfusion Organization, Bushehr, Iran; 4 Research Center for Biochemistry and Nutrition in Metabolic Diseases, Kashan University of Medical Sciences, Kashan, Iran; 5 Reproductive Health and Midwifery Department, School of Nursing and Midwifery, Shahroud University of Medical Sciences, Shahroud, Iran; Institut Pasteur, FRANCE

## Abstract

**Background:**

Blood transfusion is considered a potential risk factor for transmission of life-threatening viral infections, including HIV, HCV and HBV infections. This study was performed to find out the prevalence and trends of these infections among blood donors in Southern Iran.

**Methods:**

The blood donor data recorded in twelve regional blood transfusion centers from 2004 to 2014 were analyzed in an anonymous way with respect to the results of serological screening for HBV, HCV, and HIV infections. Overall, 293454 donors were screened for viral infections.

**Results:**

Most of the donors were male, married, aged between 20–40 years, educated, and regular donors. The overall seroprevalence rates of HBV, HCV and HIV were 0.15%, 0.1% and 0.004%, respectively. The highest seroprevalence was found for HBV, followed by HCV and HIV. These infections were more prevalent in male, low educated and first time donors. The highest HCV seroprevalence was observed among donors aged 20 to 40 years, while HBV seroprevalence increased with age. The seroprevalence rates of HBV and HCV from 2004 to 2014 showed significant decreasing trends from 0.460% to 0.060% (*P* < 0.001) and 0.329% to 0.045% (*P* < 0.001), respectively. Whereas HIV infection had a slight but not significant decline from 0.0173% in 2004 to 0.0028% in 2014 (*P* = 0.087).

**Conclusions:**

The decreasing trends of transfusion-transmissible viral infections in blood donations indicate that the attempts of IBTO were successful in improving the safety of the blood supply, since the prevalence rates of viral infections have been reduced to very low levels in blood donations over the years. However, still more effective techniques such as polymerase chain reaction (PCR) are needed to guarantee blood safety.

## Introduction

The aim of blood transfusion is protection of life, but at the same time, it can be life threatening if blood safety is not considered [1, 2]. One of the main problems in providing safe blood is the risk of transfusion-transmissible infections (TTIs). Amongst them, viral infectious agents such as hepatitis C virus (HCV), hepatitis B virus (HBV) and human immunodeficiency virus (HIV) are of the greatest concern [1, 3–5].

HIV, HBV and HCV are the causative agents of acquired immune deficiency syndrome (AIDS), hepatitis B and C infections, respectively. These infections are capable of causing long-term carrier states, prolonged viraemia and infectivity, chronic disorders along with high rates of morbidity and mortality due to chronicity, liver cirrhosis, hepatocellular carcinoma (HCC), and opportunistic infections [3, 4, 6, 7]. These viruses can be transmitted through direct exposure to infected blood and blood derivatives, organ transplantation, hemodialysis, intravenous drug use, blood transfusion, tattooing, and sexual contact [4, 6, 8]. However, the later is not the common mode of HCV transmission [8, 9].

The risk of transmission of these viruses through transfusion of infected blood is much higher than the other routes of transmission, mainly because of transmission of high viral load per transfusion [3]. Even if the viral load is low in the blood, the chance of infectivity is still very high [3]. However, currently blood transfusion has a relatively low contribution in the overall transmission of viral infections owing to this obligation that screening of blood donations for viral infections prior to transfusion is the highest priority [10].

The prevalence of these viral infections among blood donors varies by geography and nationality and directly depends on the prevalence of these viruses in the general population [11, 12]. Globally, there are approximately 170 million individuals chronically infected with HCV, 350 million with HBV, and 38 million HIV infected people [13]. According to WHO reports, the prevalence of HBV, HCV and HIV infections among blood donors in different parts of the world varies from 0.008% to 6.08%, 0.004% to 1.96%, and 0.0004% to 2.0%, respectively [14]. In Iran, the prevalence of HBV, HIV and HCV infections is 1.7% [15], 0.023% [8] and less than 1% [9] in the general population and 0.7% [16], 0.004% [8] and 0.5% [17] in blood donors, respectively.

The prevalence of any infection shows a wide range of variation in different regions of a country [9]. However, the overall prevalence of HCV among blood donors in Iran has been reported to be 0.5%, but it ranges from 0.03% to 2.1% in different parts of the country [9]. The same scenario has been reported for HBV [16]. Apart from this, the prevalence rates may change with time [2]. Variations in the donor screening strategies and the predominance of risk factors in the society might explain these changes in the prevalence rates of viral infection over time [9, 18, 19]. It is therefore necessary to assess the prevalence of these viruses among blood donors at regular intervals to estimate the current most prevalent risk factors and to evaluate the effectiveness of the blood safety strategies employed in the blood banks of a country [17, 19, 20].

Evaluation of trends in the prevalence of viral infections among blood donors is not only essential for estimating the effectiveness of the blood safety strategies [8, 18–20], but it also gives clue to health policy makers to improve the current blood bank strategies to minimize the potential risk of acquiring these infections through blood transfusion [3, 10, 21]. Therefore, this study was conducted to report the prevalence and trends of HBV, HCV and HIV infections among blood donors in south of Iran during an eleven-year period from 2004 to 2014. This is the first report on the trends of HBV, HCV and HIV infections among the donor population in this part of Iran.

## Materials and Methods

### Study setting and population

This is a retrospective descriptive study of blood donor data recorded at blood transfusion centers of southern Iran over a period of eleven years. Twelve regional blood transfusion centers are located in this part of Iran, while Bushehr blood transfusion center as the largest center contributes in collecting the most of blood units. Bushehr province, which is located in the northern shores of Persian Gulf, is a commercial port and an important economic center of southern Iran. The records of all blood donors who donated blood at one of these blood centers from 2004 to 2014 were analyzed in an anonymous way with respect to the results of serological screening for HBV, HCV, and HIV infections. This study was approved by the ethical committee of Bushehr University of Medical Sciences with reference number IR.BPUMS.Rec.1394.108.

As a routine pre-donation practice, all of donors went through a physical examination and health history interview prior to donation in an attempt to ensure their eligibility for donating blood. Those who had age between 18 and 65 years, body weight above 45 kg, hemoglobin level of 12.5 g/dL or greater, physical and mental fitness, no history of high-risk behavior, blood transfusion, jaundice, hepatitis, surgery, and hypertension, as well as the other serious illness and current fever were considered as eligible donors. Those who were outside the range of eligibility criteria were excluded.

At the time of interviewing, all donors were requested to give a written informed consent to screen their blood samples for TTIs and use of test results for analysis. All blood donors were apparently healthy volunteers and classified as first-time donors if they had a history of only one donation, regular donors if they had a history of more than one donation during one year, and repeated donors if there was more than one-year intervals between the donations.

### Screening methods

After donating blood, all donated blood was screened for the presence of hepatitis B surface antigen (HBsAg), HIV antigen/antibody (HIV Ag/Ab) and anti hepatitis C virus antibodies (anti-HCV Ab) using commercially available ELISA kits. All initially positive samples were retested. The repeatedly reactive samples were labeled seropositive. These seropositive results were confirmed using HBsAg confirmatory assay, HIV I/II Western Blot (WB), and HCV recombinant immunoblot assay (RIBA). Regarding HIV, the HIV WB-negative samples were further evaluated for the presence of HIV P24 antigen, and the reactive samples were confirmed using monoclonal neutralization assay. According to the IBTO policies, the initially positive blood units were excluded, and the confirmed positive donors were recalled for counseling and appropriate treatment. During the study period, the same kits were used in all centers (Table 1).

**Table 1:** Kits used in donor screening, 2004–2014.

**Screening test kits**			
**Year**	**HBsAg screening test kit(Manufacturer)**	**Anti-HCV screening test kit(Manufacturer)**	**HIV Ag/Ab screening test kit(Manufacturer)**
**2004**	ETI-MAK4 HBsAg(DiaSorin, Saluggia, Italy)	anti-HCV-EIA-Avicenna(Avicenna Medical Centre, Russia)	Biotest Anti-HIV-1/2 Recombinant(Biotest, Dreieich, Germany)
**2005**	ETI-MAK4 HBsAg(DiaSorin, Saluggia, Italy),Enzygnost HBsAg 5.0(Dade Behring, Marburg, Germany)	anti-HCV-EIA-Avicenna(Avicenna Medical Centre, Russia)	Biotest Anti-HIV-1/2 Recombinant(Biotest, Dreieich, Germany),Genscreen Plus HIV Ag-Ab(Bio-Rad, California, USA)
**2006**	Enzygnost HBsAg 5.0(Dade Behring, Marburg, Germany)	anti-HCV-EIA-Avicenna(Avicenna Medical Centre, Russia),HCV 3.0 with enhanced SAVe(Ortho-ClinicalDiagnostics, Inc., Raritan, USA),Hepanostica Anti-HCV Ultra(BioMerieux, Marcy l'Etoile,France)	Genscreen Plus HIV Ag-Ab(Bio-Rad, California, USA),Vironostika HIV Uni-Form II Ag/Ab(BioMerieux, Marcy l'Etoile, France)
**2007**	Enzygnost HBsAg 5.0(Dade Behring, Marburg, Germany)	HCV 3.0 with enhanced SAVe(Ortho-ClinicalDiagnostics, Inc., Raritan, USA),Hepanostica Anti-HCV Ultra(BioMerieux, Marcy l'Etoile,France)	Genscreen Plus HIV Ag-Ab(Bio-Rad, California, USA),Vironostika HIV Uni-Form II Ag/Ab(BioMerieux, Marcy l'Etoile, France)
**2008**	Enzygnost HBsAg 5.0(Dade Behring, Marburg, Germany)	HCV 3.0 with enhanced SAVe(Ortho-ClinicalDiagnostics, Inc., Raritan, USA),Hepanostica Anti-HCV Ultra(BioMerieux, Marcy l'Etoile, France)	Genscreen Plus HIV Ag-Ab (Bio-Rad, California, USA),Vironostika HIV Uni-Form II Ag/Ab(BioMerieux, Marcy l'Etoile, France)
**2009**	Enzygnost HBsAg 5.0(Dade Behring, Marburg, Germany)	HCV 3.0 with enhanced SAVe(Ortho-ClinicalDiagnostics, Inc., Raritan, USA)	Genscreen Plus HIV Ag-Ab (Bio-Rad, California, USA), Vironostika HIV Uni-Form II Ag/Ab (BioMerieux, Marcy l'Etoile, France)
**2010**	Enzygnost HBsAg 5.0(Dade Behring, Marburg, Germany),Enzygnost HBsAg 6.0(Siemens, Marburg, Germany)	HCV 3.0 with enhanced SAVe(Ortho-ClinicalDiagnostics, Inc., Raritan, USA),Hepanostica Anti-HCV Ultra(BioMerieux, Marcy l'Etoile, France)	Genscreen Plus HIV Ag-Ab(Bio-Rad, California, USA),Vironostika HIV Uni-Form II Ag/Ab(BioMerieux, Marcy l'Etoile, France)
**2011**	Enzygnost HBsAg 6.0(Siemens, Marburg, Germany)	HCV 3.0 with enhanced SAVe(Ortho-ClinicalDiagnostics, Inc., Raritan, USA), Hepanostica Anti-HCV Ultra(BioMerieux, Marcy l'Etoile, France)	EIAgen Detect HIV 4 Total Screening Kit(Adaltis Inc., Montreal, Canada)
**2012**	Enzygnost HBsAg 6.0(Siemens, Marburg, Germany)	EIAgen HCV Ab test(Adaltis Inc., Montreal, Canada)	EIAgen Detect HIV 4 Total Screening Kit(Adaltis Inc., Montreal, Canada)
**2013**	Enzygnost HBsAg 6.0(Siemens, Marburg, Germany)	EIAgen HCV Ab test(Adaltis Inc., Montreal, Canada)	EIAgen Detect HIV 4 Total Screening Kit(Adaltis Inc., Montreal, Canada)
**2014**	Enzygnost HBsAg 6.0(Siemens, Marburg, Germany)	EIAgen HCV Ab test(Adaltis Inc., Montreal, Canada)	EIAgen Detect HIV 4 Total Screening Kit(Adaltis Inc., Montreal, Canada)
**Confirmatory test kits**			
**Year**	**HBsAg confirmatory test(Manufacturer)**	**HCV RIBA(Manufacturer)**	**HIV Western Blot(Manufacturer)**
**2004**	HBsAg confirmatory test(DiaSorin, **Saluggia, Italy**)	HCV Blot 3.0(Genelabs diagnostics, Singapore)	HIV Blot 2.2(Genelabs diagnostics, Singapore)
**2005**	HBsAg confirmatory test(DiaSorin, Saluggia, Italy), HBsAg confirmatory test(Dade Behring, Marburg, Germany)	HCV Blot 3.0(Genelabs diagnostics, Singapore)	HIV Blot 2.2(Genelabs diagnostics, Singapore)
**2006**	HBsAg confirmatory test(Dade Behring, Marburg, Germany)	HCV Blot 3.0(Genelabs diagnostics, Singapore)	HIV Blot 2.2(Genelabs diagnostics, Singapore)
**2007**	HBsAg confirmatory test(Dade Behring, Marburg, Germany)	MP Diagnostics HCV BLOT 3.0(MP Biomedicals, California, USA)	HIV Blot 2.2(Genelabs diagnostics, Singapore),MP Diagnostics HIV Blot 2.2(MP Biomedicals, California, USA)
**2008**	HBsAg confirmatory test(Dade Behring, Marburg, Germany)	MP Diagnostics HCV BLOT 3.0(MP Biomedicals, California, USA)	HIV Blot 2.2(Genelabs diagnostics, Singapore),MP Diagnostics HIV Blot 2.2(MP Biomedicals, California, USA)
**2009**	HBsAg confirmatory test(Dade Behring, Marburg, Germany), HBsAg confirmatory test(Siemens, Marburg, Germany)	HCV Blot 3.0(Genelabs diagnostics, Singapore)	HIV Blot 2.2(Genelabs diagnostics, Singapore),MP Diagnostics HIV Blot 2.2 (MP Biomedicals, California, USA)
**2010**	HBsAg confirmatory test(Siemens, Marburg, Germany)	MP Diagnostics HCV BLOT 3.0(MP Biomedicals, California, USA)	MP Diagnostics HIV Blot 2.2(MP Biomedicals, California, USA)
**2011**	HBsAg confirmatory test(Siemens, Marburg, Germany)	HCV Blot 3.0(Genelabs diagnostics, Singapore)	MP Diagnostics HIV Blot 2.2(MP Biomedicals, California, USA)
**2012**	HBsAg confirmatory test(Siemens, Marburg, Germany)	MP Diagnostics HCV BLOT 3.0(MP Biomedicals, California, USA)	MP Diagnostics HIV Blot 2.2(MP Biomedicals, California, USA)
**2013**	HBsAg confirmatory test(Siemens, Marburg, Germany)	MP Diagnostics HCV BLOT 3.0(MP Biomedicals, California, USA)	MP Diagnostics HIV Blot 2.2(MP Biomedicals, California, USA)
**2014**	HBsAg confirmatory test(Siemens, Marburg, Germany)	MP Diagnostics HCV BLOT 3.0(MP Biomedicals, California, USA)	MP Diagnostics HIV Blot 2.2(MP Biomedicals, California, USA)

### Statistical analysis

Data were entered in Microsoft Excel sheets and analyzed using OpenEpi statistical software (Available at http://openepi.com/Menu/OE_Menu.htm). All data were provided as frequencies and percentages for basic descriptive purposes. The yearly seroprevalence rates of TTIs were presented for the entire study population and different socio-demographic categories. Chi-square test was used to compare the seroprevalence rates of TTIs among blood donors grouped according to socio-demographic characteristics and evaluate the impact of categorical variables on TTIs seropositivity. Chi-square test for trend was used to analyze the variations in trends of TTIs during this eleven-year period. Statistically, *P* values of less than 0.05 were accepted as significant.

## Results

During the 11-year period from 2004 through 2014, 376568 individuals volunteered to donate blood. Of them, 293454 (77.93%) donors were screened for viral infections, and 83114 (22.07%) candidates were deferred by the pre-donation questionnaire. A gradual increase in the number of donors was observed over time from 1337.4 donors per 100,000 population in 2004 to 3310.8 donors per 100,000 population in 2014. Out of 293454 donors, 274556 (93.5%) were males and 18898 (6.5%) were females, 239211 (81.5%) were married while 54243 (18.5%) were single. Regular donors represented the majority of donors, followed by first time donors and repeated donors. A considerable percentage of the donors were educated and a few illiterate, 62.5% had diploma or higher level of education. The age groups 20–30 years and 31–40 years contributed a large percentage of all donations. The majority of blood donations (84.5%) were obtained from Bushehr and Borazjan cities, while the remaining donations (15.5%) were collected from the other cities. Private business owners, clerical workers and military personnel constituted the majority of donors. The socio-demographic characteristics of donors, including gender, marital status, type of donor, education level, age, place of residency and profession are summarized in Table 2.

**Table 2:** Socio-demographic characteristics of blood donors in south of Iran, 2004–2014.

	2004	2005	2006	2007	2008	2009	2010	2011	2012	2013	2014	Total
Number of donors	11,521	17,735	19,073	25,573	28,065	26,509	28,936	32,952	33,524	34,586	34,980	293,454
**Gender**N(%)												
Male	10,366(90.0)	16,097(90.8)	17,549(92.0)	23,613(92.3)	26,325(93.8)	24,903(94.0)	27,126(93.8)	30,936(93.9)	31,593(94.2)	32,841(95.0)	33,207(95.0)	274,556(93.5)
Female	1,155(10.0)	1,638(9.2)	1,524(8.0)	1,960(7.7)	1,740(6.2)	1,606(6.0)	1,810(6.2)	2,016(6.1)	1,931(5.8)	1,745(5.0)	1,773(5.0)	18,898(6.5)
**Marital status**N(%)												
Single	2,634(22.9)	3,679(20.7)	3,628(19.0)	5,181(20.3)	5,288(18.8)	4,723(17.8)	5,475(19.0)	5,691(17.3)	5,937(17.7)	6,074(17.5)	5,933(17.0)	54,243(18.5)
Married	8,887(77.1)	14,056(79.3)	15,445(81.0)	20,392(79.7)	22,777(81.2)	21,786(82.2)	23,461(81.0)	27,261(82.7)	27,587(82.3)	28,512(82.5)	29,047(83.0)	239,211(81.5)
**Type of blood donor** N(%)												
First-time donor	9,799(85.05)	10,409(58.7)	8421(44.1)	11,959(46.8)	10,351(36.9)	8,392(31.7)	8,666(30.0)	8,473(25.7)	7,895(23.6)	7,700(22.3)	6,708(19.2)	98,773(33.6)
Repeated donor	6(0.05)	626(3.5)	2,645(13.9)	3,995(15.6)	4,944(17.6)	5,896(22.2)	6,199(21.4)	6,795(20.6)	7,583(22.6)	7,974(23.0)	8,450(24.2)	55,113(18.8)
Regular donor	1,716(14.9)	6,700(37.8)	8,007(42.0)	9,619(37.6)	12,770(45.5)	12,221(46.1)	14,071(48.6)	17,684(53.7)	18,046(53.8)	18,912(54.7)	19,822(56.6)	139,568(47.6)
**Level of education**N(%)												
Illiterate	411(3.6)	601(3.4)	589(3.08)	923(3.6)	894(3.2)	841(3.2)	815(2.8)	908(2.7)	822(2.5)	749(2.2)	754(2.1)	8,307(2.8)
Under diploma	3,918(34.0)	6,198(34.9)	6,979(36.6)	9,465(37.0)	9,912(35.3)	9,327(35.2)	9,995(34.6)	11,362(34.5)	11,001(32.8)	11,568(33.4)	11,592(33.1)	101,317(34.6)
Diploma	5,015(43.5)	7,573(42.7)	7,761(40.7)	10,232(40.0)	11,405(40.7)	10,983(41.4)	11,880(41.0)	13,041(39.6)	13,455(40.1)	13,412(38.8)	13,557(38.8)	118,314(40.3)
Higher diploma	2,177(18.9)	3,363(19.0)	3,738(19.6)	4,918(19.3)	5,704(20.3)	5,270(19.9)	6,193(21.4)	7,640(23.2)	8,246(24.6)	8,857(25.6)	9,077(26.0)	65,183(22.2)
No record	0(0.0)	0(0.0)	6(0.03)	35(0.14)	150(0.53)	88(0.33)	53(0.2)	1(0.003)	0(0.0)	0(0.0)	0(0.0)	333(0.11)
**Age groups(years)** N(%)												
<20	1,180(10.2)	2,241(12.6)	3,698(19.4)	2,444(9.6)	1,767(6.3)	1,384(5.2)	1,326(4.6)	1,198(3.6)	1,017(3.0)	837(2.4)	703(2.0)	17,795(6.0)
20–30	4,313(37.4)	6,409 (36.1)	6,331(33.2)	9,297(36.3)	10,658(38.0)	9,677(36.5)	10,320(35.6)	11,584(35.2)	11,411(34.0)	11,185(32.3)	10,362(29.6)	101,547(34.6)
31–40	3,340(29.0)	5,008(28.2)	4,979(26.1)	7,499(29.3)	8,734(31.1)	8,530(32.2)	9,367(32.4)	10,813(32.8)	11,304(33.8)	12,198(35.3)	12,902(36.9)	94,674(32.3)
41–50	1,968(17.1)	2,952(16.7)	2,938(15.4)	4,495(17.6)	4,978(17.7)	4,903(18.5)	5,555(19.2)	6,527(19.8)	6,839(20.4)	7,326(21.2)	7,871(22.5)	56,352(19.2)
51–60	667(5.8)	1,008(5.7)	1,035(5.4)	1,708(6.7)	1,786(6.4)	1,895(7.2)	2,221(7.7)	2,665(8.1)	2,739(8.2)	2,853(8.3)	3,007(8.6)	21,584(7.4)
>60	53(0.5)	117(0.7)	92(0.5)	130(0.5)	142(0.5)	120(0.4)	147(0.5)	165(0.5)	214(0.6)	187(0.5)	135(0.4)	1,502(0.5)
**Place of residence**N(%)												
Bushehr	10,275(89.2)	13,472(76.0)	18,736(98.2)	17,816(69.7)	15,788(56.3)	15,248(57.5)	16,753(57.9)	19,347(58.7)	20,001(59.7)	19,899(57.5)	20,768(59.4)	188,103(64.1)
Genaveh	617(5.4)	1,630(9.2)	133(0.7)	298(1.2)	1,237(4.4)	2,177(8.2)	2,121(7.3)	2,306(7.0)	2,574(7.7)	2,406(7.0)	2,572(7.3)	18,071(6.2)
Borazjan	59(0.5)	1(0.006)	0(0.0)	5,623(22.0)	7,254(25.9)	6,200(23.4)	7,329(25.3)	8,189(24.9)	8,089(24.1)	8,642(25.0)	8,629(24.7)	60,015(20.4)
Khormuj	162(1.4)	401(2.3)	72(0.4)	339(1.3)	756(2.7)	556(2.1)	474(1.6)	276(0.84)	397(1.2)	245(0.70)	299(0.85)	3,977(1.3)
Khark	101(0.9)	532(3.0)	38(0.2)	290(1.1)	860(3.0)	499(1.9)	190(0.66)	322(0.98)	247(0.74)	279(0.80)	340(0.97)	3,698(1.3)
Ahram	65(0.6)	350(2.0)	31(0.16)	370(1.4)	420(1.5)	364(1.4)	204(0.70)	98(0.30)	269(0.80)	81(0.23)	121(0.34)	2,373(0.80)
Jam	155(1.3)	650(3.6)	50(0.3)	459(1.8)	760(2.7)	559(2.1)	499(1.7)	320(0.97)	433(1.3)	339(0.98)	371(1.06)	4,595(1.6)
Dayer	38(0.3)	127(0.7)	3(0.02)	76(0.3)	210(0.74)	227(0.85)	108(0.4)	147(0.45)	89(0.26)	79(0.23)	65(0.19)	1,169(0.4)
Kangan	0(0.0)	359(2.02)	0(0.0)	198(0.8)	488(1.73)	459(1.73)	1,034(3.6)	1821(5.5)	1216(3.6)	2526(7.3)	1610(4.6)	9,711(3.3)
Deylam	34(0.3)	143(0.8)	0(0.0)	103(0.4)	240(0.85)	147(0.55)	101(0.35)	87(0.26)	136(0.40)	61(0.18)	76(0.22)	1,128(0.4)
Tange eram	10(0.09)	40(0.22)	3(0.02)	0(0.0)	34(0.12)	45(0.17)	86(0.30)	23(0.07)	44(0.13)	25(0.07)	87(0.25)	397(0.13)
Abpakhsh	5(0.04)	30(0.17)	7(0.04)	1(0.004)	18(0.06)	28(0.10)	37(0.13)	16(0.05)	29(0.09)	4(0.01)	42(0.12)	217(0.07)
**Occupation**N(%)												
Private business owner	4,209(36.6)	7,325(41.3)	8,398(44.0)	11,497(45.0)	13,117(46.8)	12,692(47.9)	14,097(48.7)	15,920(48.3)	16,238(48.4)	17,470(50.5)	17,856(51.0)	138,819(47.3)
Clerical worker	3,466(30.1)	4,953(27.9)	5,190(27.2)	6,960(27.2)	7,198(25.7)	6,590(24.9)	6,938(24.0)	7,703(23.4)	7,935(23.7)	7,864(22.7)	7,872(22.5)	72,669(24.8)
Homemaker	1,028(8.9)	1,506(8.5)	1,488(7.8)	1,813(7.1)	1,640(5.8)	1,468(5.5)	1,543(5.3)	1,812(5.5)	1,648(4.9)	1,515(4.4)	1,562(4.5)	17,023(5.8)
Military personnel	1,676(14.6)	2,268(12.8)	2,076(10.9)	2,917(11.4)	3,319(11.8)	3,052(11.5)	3,310(11.4)	3,774(11.5)	3,736(11.14)	3,621(10.5)	3,726(10.7)	33,475(11.4)
Retiree	124(1.08)	221(1.2)	250(1.3)	335(1.3)	303(1.08)	342(1.3)	436(1.5)	619(1.9)	618(1.84)	587(1.7)	603(1.7)	4,438(1.5)
Pupil	124(1.08)	182(1.03)	208(1.1)	316(1.2)	310(1.1)	388(1.5)	419(1.5)	357(1.08)	344(1.03)	318(0.92)	299(0.85)	3,265(1.1)
University student	497(4.3)	762(4.3)	844(4.4)	927(3.6)	1,189(4.2)	1,121(4.2)	1,299(4.5)	1,369(4.15)	1,412(4.2)	1,473(4.2)	1,344(3.8)	12,237(4.16)
Conscript	212(1.8)	245(1.4)	354(1.9)	385(1.5)	512(1.8)	372(1.4)	326(1.12)	291(0.88)	245(0.73)	266(0.77)	301(0.86)	3,509(1.2)
Unemployed	153(1.3)	244(1.4)	238(1.2)	359(1.4)	445(1.6)	435(1.6)	513(1.8)	805(2.4)	956(2.9)	996(2.9)	902(2.6)	6,046(2.06)
Others	32(0.28)	29(0.16)	27(0.14)	64(0.25)	32(0.11)	49(0.18)	55(0.19)	302(0.91)	392(1.17)	476(1.4)	515(1.5)	1,973(0.67)

Of 293454 donors, 747 were determined to be seropositive for any of the screened TTIs, revealing an overall seroprevalence rate of 0.254% for TTIs. Overall, the seroprevalence rates of HBV, HCV and HIV were 0.15% (440), 0.1% (295) and 0.004% (12), respectively. None of the blood donors had co-infection with these viruses.

The seroprevalence of HCV, HIV and HBV infections in male donors was 0.104%, 0.004% and 0.150%, respectively, and the corresponding figures for females were 0.047%, 0% and 0.137%, respectively. Although HBV and HCV infections were more prevalent among male donors compared to the females, but only difference in the prevalence of HCV was statistically significant (*P* = 0.017). Regarding HIV infection, no seropositive case was found among the female donors.

The seroprevalence of HCV and HIV were higher among single donors, whereas HBV infection was more prevalent among married donors. The seroprevalence of HCV, HIV and HBV infections among single donors was 0.132%, 0.007% and 0.114%, respectively. While the corresponding figures among married donors were 0.093%, 0.003% and 0.158%, respectively. No significant difference in HIV prevalence was observed between the donors regarding marital status, while the differences in the prevalence of HBV and HCV were statistically significant (*P* = 0.017 and *P* = 0.008, respectively). Compared to regular and repeated donors, first-time donors were significantly more infected by HBV (*P* < 0.001), HCV (*P* < 0.001) and HIV (*P* = 0.001), while the frequency of these three pathogens was the lowest in regular donors.

The seroprevalence of TTIs decreased with education level, so that highly educated donors showed lower seropositivity rates. Overall, there were significant differences in HBV and HCV seroprevalence rates between the donors grouped according to level of education (*P* < 0.001, *P* < 0.001, respectively). For HIV, a small but not significant difference was observed between donors with diploma and under diploma education levels (*P* = 0.173).

There were significant differences in the seroprevalence of HBV and HCV between the age groups (*P* = 0.014 and *P* = 0.005, respectively). HBV seroprevalence increased with age, from 0.089% in donors under 20 years old to 0.399% in donors over 60 years old. As for HCV, the highest rate of HCV seroprevalence was observed in the age group 31–40 years followed by age group 20–30 years. While donors in the age group 51–60 years showed the lowest positivity rate.

HIV seropositivity was only observed in the age groups 20–30 and 31–40 years. However, HIV was more prevalent in donors aged 20–30 years, but this difference was not significant (*P* = 0.183).

HBV seropositivity was observed among donor populations of all the cities, while no positive HCV case was found in blood donors from Abpakhsh city. In addition, HIV seroprevalence was only observed in Bushehr, Genaveh and Borazjan cities, and no significant difference was reported among these cities (*P* = 0.997). Meanwhile, significant differences in the seroprevalence of HBV (*P* = 0.004) and HCV (*P* < 0.001) were observed between the donors regarding the place of residency. The highest seroprevalence rates of HBV and HCV were observed among donors in Abpakhsh and Tange Eram cities, respectively. While Genaveh and Jam cities had the lowest rates of hepatitis B and C among blood donors in this region, respectively.

There were statistical differences in the prevalence rates of HBV (*P* = 0.021) and HCV (*P* < 0.001) between the donors grouped according to their occupation. Conscripts (0.227%) and retirees (0.225%) had the highest seroprevalence rates for HBV, while unemployed (0.215%) displayed the highest seroprevalence for HCV. On the other hand, university students (0.049%) and retirees (0.022%) showed the lowest seroprevalence rates for HBV and HCV infections, respectively. However, HIV seroprevalence was higher among private business owners, it was not statistically associated with working status (*P* = 0.379). The yearly distribution of HBV, HCV and HIV seropositivity among donors grouped according to socio-demographic characteristics is shown in Tables 3–5, respectively.

**Table 3:** Yearly seroprevalence of HBV infection among blood donors according to socio-demographic characteristics.

	2004	2005	2006	2007	2008	2009	2010	2011	2012	2013	2014	Total	*P* value
**HBV positive**	53(0.460)	70(0.394)	48(0.251)	53(0.207)	44(0.156)	46(0.173)	30(0.103)	28(0.084)	25(0.074)	22(0.063)	21(0.060)	440(0.149)	**<0.001**
**Gender**N(%)													**0.649**
Male	51(0.491)	66(0.410)	46(0.262)	50(0.211)	40(0.151)	44(0.176)	29(0.106)	27(0.087)	23(0.072)	20(0.060)	18(0.054)	414(0.150)	
Female	2(0.173)	4(0.244)	2(0.131)	3(0.153)	4(0.229)	2(0.124)	1(0.055)	1(0.049)	2(0.103)	2(0.114)	3(0.169)	26(0.137)	
**Marital status**N(%)													**0.017**
Single	7(0.265)	5(0.135)	6(0.165)	10(0.193)	7(0.132)	7(0.148)	6(0.109)	2(0.035)	5(0.084)	4(0.065)	3(0.050)	62(0.114)	
Married	46(0.517)	65(0.462)	42(0.271)	43(0.210)	37(0.162)	39(0.179)	24(0.102)	26(0.095)	20(0.072)	18(0.063)	18(0.061)	378(0.158)	
**Type of blood donors**N(%)													**<0.001**
First-time donor	50(0.510)	63(0.605)	39(0.463)	51(0.426)	42(0.405)	43(0.512)	30(0.346)	25(0.295)	23(0.291)	20(0.259)	19(0.283)	405(0.410)	
Repeated donor		1(0.159)	5(0.189)	1(0.025)		3(0.050)		1(0.014)	1(0.013)		2(0.023)	14(0.025)	
Regular donor	3(0.174)	6(0.089)	4(0.049)	1(0.010)	2(0.015)			2(0.011)	1(0.005)	2(0.010)		21(0.015)	
**Level of education**N(%)													**<0.001**
Illiterate	1(0.243)	4(0.665)	4(0.679)	4(0.433)	3(0.335)	1(0.118)	2(0.245)	6(0.660)			3(0.397)	28(0.337)	
Under diploma	13(0.331)	23(0.371)	27(0.386)	28(0.295)	26(0.262)	25(0.268)	14(0.140)	10(0.088)	15(0.136)	10(0.086)	11(0.094)	202(0.199)	
Diploma	27(0.538)	33(0.435)	13(0.167)	19(0.185)	8(0.070)	15(0.136)	9(0.075)	9(0.069)	6(0.044)	5(0.037)	3(0.022)	147(0.124)	
Higher diploma	12(0.551)	10(0.297)	4(0.107)	2(0.040)	7(0.122)	5(0.094)	5(0.080)	3(0.039)	4(0.048)	7(0.079)	4(0.044)	63(0.096)	
**Age groups(years)**N(%)													**0.014**
<20	5(0.423)	3(0.133)	1(0.027)	4(0.163)		2(0.144)					1(0.142)	16(0.089)	
20–30	18(0.417)	23(0.358)	22(0.347)	17(0.182)	16(0.150)	16(0.165)	15(0.145)	8(0.069)	8(0.070)	6(0.053)	3(0.028)	152(0.149)	
31–40	16(0.479)	25(0.499)	17(0.341)	12(0.160)	12(0.137)	15(0.175)	8(0.085)	8(0.073)	7(0.061)	5(0.040)	6(0.046)	131(0.138)	
41–50	11(0.558)	16(0.542)	6(0.204)	10(0.222)	8(0.160)	12(0.244)	5(0.090)	6(0.091)	8(0.116)	8(0.109)	8(0.101)	98(0.173)	
51–60	3(0.449)	3(0.297)	2(0.193)	9(0.526)	4(0.223)	1(0.052)	1(0.045)	6(0.225)	2(0.073)	3(0.105)	3(0.099)	37(0.171)	
>60		1(0.854)	1(1.086)	1(0.769)	2(1.408)		1(0.680)					6(0.399)	
**Place of residence**N(%)													**0.004**
Bushehr	49(0.476)	64(0.475)	48(0.256)	43(0.241)	26(0.164)	25(0.163)	14(0.083)	15(0.077)	15(0.074)	11(0.055)	7(0.033)	317(0.168)	
Borazjan				10(0.177)	9(0.124)	10(0.161)	5(0.068)	6(0.073)	9(0.111)	7(0.081)	9(0.104)	65(0.108)	
Kangan					1(0.204)	1(0.217)	6(0.580)	5(0.274)		3(0.118)	5(0.310)	21(0.216)	
Genaveh	1(0.162)	6(0.368)			2(0.161)	1(0.045)	1(0.047)		1(0.038)			12(0.066)	
Khormuj	3(1.851)				1(0.132)	1(0.179)	2(0.421)					7(0.176)	
Jam					1(0.131)	2(0.357)	2(0.400)			1(0.294)		6(0.130)	
Ahram					1(0.238)	2(0.549)						3(0.126)	
Dayer						1(0.440)		2(1.360)				3(0.256)	
Khark					2(0.232)	1(0.200)						3(0.081)	
Deylam					1(0.416)							1(0.088)	
Tange eram						1(2.222)						1(0.251)	
Abpakhsh						1(3.571)						1(0.460)	
**Occupation**N(%)													**0.021**
Private business owner	14(0.332)	34(0.464)	29(0.345)	37(0.321)	24(0.182)	28(0.220)	21(0.148)	15(0.094)	14(0.086)	7(0.040)	8(0.044)	231(0.166)	
Clerical worker	19(0.548)	18(0.363)	11(0.211)	7(0.100)	9(0.125)	8(0.121)	7(0.100)	6(0.077)	4(0.050)	10(0.127)	7(0.088)	106(0.145)	
University student	1(0.201)	2(0.262)		1(0.107)	2(0.168)							6(0.049)	
Homemaker	2(0.194)	4(0.265)	2(0.134)	3(0.165)	3(0.182)	2(0.136)	1(0.064)	1(0.055)	2(0.121)	2(0.132)	3(0.192)	25(0.146)	
Military personnel	11(0.656)	10(0.440)	3(0.144)	3(0.102)	3(0.090)	4(0.131)		4(0.105)	3(0.080)			41(0.122)	
Unemployed	2(1.307)	1(0.409)		1(0.278)				2(0.248)	1(0.104)	2(0.200)	1(0.110)	10(0.165)	
Retiree	2(1.612)		1(0.4)	1(0.298)	2(0.660)	1(0.292)	1(0.229)		1(0.161)		1(0.165)	10(0.225)	
Pupil		1(0.549)		1(0.316)		1(0.257)						3(0.091)	
Conscript	2(0.943)		2(0.564)			2(0.537)				1(0.375)	1(0.332)	8(0.227)	

**Table 4:** Yearly seroprevalence of HCV infection among blood donors according to socio-demographic characteristics.

	2004	2005	2006	2007	2008	2009	2010	2011	2012	2013	2014	Total	*P* value
**HCV positive**	38(0.329)	42(0.236)	41(0.214)	43(0.168)	25(0.089)	28(0.105)	18(0.062)	10(0.030)	18(0.053)	16(0.046)	16(0.045)	295(0.100)	**<0.001**
**Gender**N(%)													**0.017**
Male	37(0.356)	42(0.260)	40(0.227)	42(0.177)	24(0.091)	27(0.108)	18(0.066)	10(0.032)	16(0.050)	14(0.042)	16(0.048)	286(0.104)	
Female	1(0.086)		1(0.065)	1(0.051)	1(0.057)	1(0.062)			2(0.103)	2(0.114)		9(0.047)	
**Marital status**N(%)													**0.008**
Single	13(0.493)	12(0.326)	10(0.275)	12(0.231)	4(0.075)	6(0.127)	2(0.036)	2(0.035)	4(0.067)	3(0.049)	4(0.067)	72(0.132)	
Married	25(0.281)	30(0.213)	31(0.200)	31(0.152)	21(0.092)	22(0.100)	16(0.068)	8(0.029)	14(0.050)	13(0.045)	12(0.041)	223(0.093)	
**Type of blood donors**N(%)													**<0.001**
First-time donor	38(0.387)	36(0.345)	37(0.439)	37(0.309)	22(0.212)	28(0.333)	17(0.196)	10(0.118)	13(0.164)	15(0.194)	12(0.178)	265(0.268)	
Repeated donor			4(0.151)	5(0.125)	2(0.040)				4(0.052)	1(0.012)	2(0.023)	18(0.032)	
Regular donor		6(0.089)		1(0.010)	1(0.007)		1(0.007)		1(0.005)		2(0.010)	12(0.008)	
**Level of education**N(%)													**<0.001**
Illiterate	3(0.729)		1(0.169)				1(0.122)			1(0.133)		6(0.072)	
Under diploma	23(0.587)	28(0.451)	21(0.300)	30(0.316)	14(0.141)	18(0.192)	14(0.140)	5(0.044)	10(0.090)	8(0.069)	7(0.060)	178(0.175)	
Diploma	10(0.199)	9(0.118)	15(0.193)	10(0.097)	8(0.070)	8(0.072)		4(0.030)	6(0.044)	5(0.037)	7(0.051)	82(0.069)	
Higher diploma	2(0.091)	5(0.148)	4(0.107)	3(0.061)	3(0.052)	2(0.037)	3(0.048)	1(0.013)	2(0.024)	2(0.022)	2 (0.022)	29(0.044)	
**Age groups(years)**N(%)													**0.005**
<20	2(0.169)	2(0.089)	2(0.054)	1(0.040)	2(0.113)						1(0.142)	10(0.056)	
20–30	19(0.440)	21(0.327)	18(0.284)	16(0.172)	10(0.093)	8(0.082)	2(0.019)	2(0.017)		3(0.026)	5(0.048)	104(0.102)	
31–40	8(0.239)	14(0.279)	18(0.361)	18(0.240)	9(0.103)	15(0.175)	10(0.106)	3(0.027)	11(0.097)	8(0.065)	7(0.054)	121(0.127)	
41–50	4(0.203)	5(0.169)	2(0.068)	7(0.155)	4(0.080)	5(0.101)	6(0.108)	4(0.061)	4(0.058)	3(0.040)	3(0.038)	47(0.083)	
51–60	4(0.599)		1(0.096)	1(0.058)				1(0.037)	3(0.109)	2(0.070)		12(0.055)	
>60	1(1.886)											1(0.066)	
**Place of residence**N(%)													**<0.001**
Bushehr city	34(0.330)	39(0.289)	41(0.218)	43(0.241)	15(0.095)	12(0.078)	11(0.065)	4(0.020)	12(0.059)	12(0.060)	13(0.062)	236(0.125)	
Borazjan					6(0.082)	8(0.129)	1(0.013)	1(0.012)	3(0.037)	2(0.023)	3(0.034)	24(0.039)	
Genaveh	3(0.486)	3(0.184)			2(0.161)	5(0.229)	2(0.094)	1(0.043)	2(0.077)	2(0.083)		20(0.110)	
Khark					1(0.116)			2(0.621)				3(0.081)	
Ahram						1(0.274)	1(0.490)		1(0.371)			3(0.126)	
Kangan							1(0.096)	2(0.109)				3(0.030)	
Khormuj	1(0.617)				1(0.132)							2(0.050)	
Jam						1(0.178)						1(0.021)	
Dayer						1(0.440)						1(0.085)	
Deylam							1(0.990)					1(0.088)	
Tange eram							1(1.162)					1(0.251)	
**Occupation**N(%)													**<0.001**
Private business owner	30(0.712)	30(0.409)	30(0.357)	31(0.269)	19(0.144)	23(0.181)	14(0.099)	8(0.050)	12(0.073)	8(0.045)	9(0.050)	214(0.154)	
Clerical worker	5(0.144)	6(0.121)	6(0.115)	5(0.071)	2(0.027)	2(0.030)	3(0.043)	1(0.012)	2(0.025)	3(0.038)	4(0.050)	39(0.053)	
University student		1(0.131)	1(0.118)		1(0.084)					1(0.067)	1(0.074)	5(0.040)	
Homemaker	1(0.097)		1(0.067)	1(0.055)		1(0.068)			1(0.060)	2(0.132)		7(0.041)	
Military personnel	2(0.119)	2(0.088)	1(0.048)	2(0.068)	1(0.030)				1(0.026)		1(0.026)	10(0.029)	
Unemployed		2(0.819)	2(0.840)	3(0.835)	1(0.224)	1(0.229)	1(0.194)	1(0.124)	2(0.209)			13(0.215)	
Retiree										1(0.170)		1(0.022)	
Pupil					1(0.322)							1(0.030)	
Conscript		1(0.408)		1(0.259)						1(0.375)	1(0.332)	4(0.113)	
Others						1(2.040)						1(0.050)	

**Table 5:** Yearly seroprevalence of HIV infection among blood donors according to socio-demographic characteristics.

	2004	2005	2006	2007	2008	2009	2010	2011	2012	2013	2014	Total	*P* value
**HIV positive**	2(0.0173)	3(0.0169)	0	0	1(0.0035)	1(0.0037)	1(0.0034)	1(0.0030)	0	2(0.0057)	1(0.0028)	12(0.0040)	**0.087**
**Gender**N(%)													**0.449**
Male	2(0.0192)	3(0.0186)			1(0.0037)	1(0.0040)	1(0.0036)	1(0.0032)		2(0.0060)	1(0.0030)	12(0.0043)	
Female													
**Marital status**N(%)													**0.220**
Single	1(0.0379)	1(0.0271)					1(0.0182)			1(0.0164)		4(0.0073)	
Married	1(0.0112)	2(0.0142)			1(0.0043)	1(0.0045)		1(0.0036)		1(0.0035)	1(0.0034)	8(0.0033)	
**Type of blood donors**N(%)													**0.001**
First-time donor	2(0.0204)	3(0.0288)			1(0.0096)	1(0.0119)	1(0.0115)	1(0.0118)		1(0.0129)		10(0.0101)	
Repeated donor										1(0.0125)		1(0.0018)	
Regular donor											1(0.0050)	1(0.0007)	
**Level of education**N(%)													**0.173**
Illiterate													
Under diploma	2(0.0510)	3(0.0484)			1(0.0100)		1(0.0100)					7(0.0069)	
Diploma						1(0.0091)		1(0.0076)		2(0.0149)	1(0.0073)	5(0.0042)	
Higher diploma													
**Age groups(years)**N(%)													**0.183**
<20													
20–30	2(0.0463)	1(0.0156)				1(0.0103)	1(0.0096)	1(0.0086)		2(0.0178)		8(0.0078)	
31–40		2(0.0399)			1(0.0114)						1(0.0077)	4(0.0042)	
41–50													
51–60													
>60													
**Place of residence**N(%)													**0.997**
Bushehr	2(0.0194)	3(0.0222)				1(0.0065)		1(0.0051)				7(0.0037)	
Genaveh											1(0.0388)	1(0.0055)	
Borazjan					1(0.0137)		1(0.0136)			2(0.0231)		4(0.0066)	
**Occupation**N(%)													**0.379**
Private business owner	2(0.0475)	3(0.0409)			1(0.0076)		1(0.0070)	1(0.0062)		2(0.0114)	1(0.0056)	11(0.0079)	
Clerical worker						1(0.0151)						1(0.0013)	

**Fig 1:**
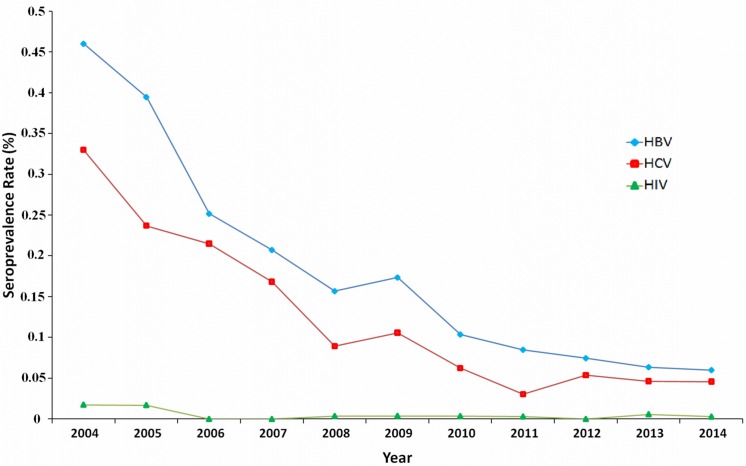
Trends of HBV, HCV and HIV infections among blood donors in south of Iran, 2004–2014.

Overall, TTIs were more prevalent in male, low educated and first-time donors during the study period. Over this 11-year period, seroprevalence rates of HBV and HCV showed significant decreasing trends from 0.460% to 0.060% (*P* < 0.001) and 0.329% to 0.045% (*P* < 0.001), respectively. Whereas prevalence of HIV infection had a slight but not significant decline from 0.0173% in 2004 to 0.0028% in 2014 (*P* = 0.087). The trends of HBV, HCV and HIV infections in all blood donations from 2004 to 2014 are shown in Fig 1. Overall, the most prevalent TTIs were HBV followed by HCV, while the prevalence of HIV infection was the lowest in southern Iran.

## Discussion

Blood transfusion is considered as a potential risk factor for transmission of life-threatening viruses, including mainly HBV, HCV and HIV [1, 5]. Therefore, efficient strategies should be implemented to reduce this risk to the minimum. Prevention of these infections is achievable by continuous screening of all blood donations for these viruses [1, 10, 17, 22]. According to the epidemiological reports, the risk of transmission of viral infections has been noticeably decreased in countries where continuous screening of all blood donations for TTIs is carried out [8, 17, 21]. In Iran, the serological screening of all donated blood is mandatory for HBV, HCV and HIV [8, 17, 19, 20]. In recent years, Iranian Blood Transfusion Organization (IBTO) has implemented more restrictive donor selection criteria through application of strict and standard questionnaire, confidential unit exclusion, and effective procedures in physical examination prior to donation as well as educational program regarding blood donation to improve the blood safety [8, 9, 17, 19, 20]. As a result, elimination or reduction of the risk of transmitting viral infection through blood transfusion has been predicted. In addition, public awareness regarding the general prevalence and transmission routes of viral infections has a considerable effect on the rates of these infections in donor population [8, 9, 17, 19, 20]. Monitoring the prevalence trends of viral infections in the donor population is a valuable index for evaluating the effectiveness of strategies implemented by IBTO [8, 17, 19, 20]. Therefore, the current study was conducted with the objective to find out the prevalence and trends of HBV, HCV and HIV infections in all blood donations from 2004 to 2014.

The results of this study demonstrate very low rates of viral infections in blood donations in Southern Iran. The seroprevalence rate of 0.15% for HBV observed in the present study is lower than those reported in previous studies from Iran, 3.4% in 1979 [23], 1.79% in 1998 [20], 0.6% in 2003–2005 [18], 0.56% in 2004–2007 [8], 0.41% in 2007 [20], 0.23% in 2009 [24], and 0.38% in 2005–2011 [19]. This is probably because of population differences regarding social behavior, lifestyle, socioeconomic status and level of awareness in different regions of our country. However, some differences in specificity and sensitivity of screening tests, number of first-time donors, and geographical distribution of the infection as well as the burden of the disease in the society can also explain a part of these variations. The reported prevalence in this study is also lower than those of Mangalore (0.5%) [1], Ethiopia (4.7%) [25], China (0.87%) [21], Mongolia (8.1%) [26], Pakistan (1.46%-2.99%) [22], Nepal (0.47%) [2], and Nigeria (11.1%) [6] but higher than those of Canada (0.007%-0.06%) [27], Australia (0.01%) [28], and Italy (0.0069%) [29]. Generally, the prevalence of HBV infection among blood donors is low in developed countries, while it is higher in developing countries. These geographical differences in prevalence of HBV infection might be due to some differences in socioeconomic status, health behaviors and attitudes, standard of life, risk behaviors, rate of this infection in the general population, immunization status, public education, level of safety measures in public health services, effectiveness of donor selection program, and quality of blood screening tests in different parts of the world.

Our findings also indicate that older donors are more prevalent infected by HBV than younger donors. This increasing prevalence with age may be associated with increased exposure to HBV most likely due to the possibility of engaging more in risky behaviors over time [4, 30]. Risky sexual activities like multiple sex partnerships more efficiently transmit HBV than HCV and HIV, while HCV transmission by sexual intercourse is less common [31–33]. In contrast, HCV is efficiently transmitted by injecting drug use, which is more common among youths in Iran [9]. Furthermore, among blood borne and sexually transmitted viruses, HBV has a low infectious dose, thus making high risk non-vaccinated population more likely to get infected [34]. In fact, initiation of HBV vaccination program for all newborns in Iran in 1993 as well as vaccination of teenagers and high-risk groups since 2006 also had an important role in decreasing the prevalence of HBV infection among youths [16]. This is consistent with previous studies, which have demonstrated a considerable association between higher HBV seroprevalence and older ages [4, 8, 21, 27, 30]. While some other studies have shown higher prevalence among younger age groups [1, 3, 6, 25, 35]. Differences in risk factors, immunization status, cultural practices such as circumcision, tattooing and phlebotomy, as well as negative social behaviors such as intravenous drug use, multiple sex relationships and history of imprisonment among different age groups in different parts of the world may explain this discrepancy among different studies.

The overall seroprevalence of 0.1% for HCV noted in our study is almost similar to the previous reports from Iran, 0.1% in 2003–2005 [18], 0.13% in 2004–2007 [8], 0.17% in 2009 [24], and 0.11% in 2005–2011 [19]. This prevalence is lower than those of the other countries such as Pakistan (3.01%-4.99%) [22], Ethiopia (0.7%) [25], China (0.86%) [21], Mongolia (8.7%) [26], Egypt (5%-25%) [36], Nepal (0.64%) [2], and Nigeria (1.8%) [6] but higher than those of Mangalore (0.08%) [1], Australia (0.01%) [28], and Italy (0.0016%) [29]. These variations in prevalence of HCV infection in different parts of the world reflect population risks, health status, rates of high-risk behaviors, public awareness, as well as quality of donor screening and selection procedure in those regions. The highest HCV seroprevalence was observed among donors aged 20 to 40 years. This can be related to high rate of injecting drug abuse among youths in Iran. Injecting drug use is the most predominant risk factor for acquiring HCV infection in our country [9, 17, 18]. Currently, the prevalence of HCV infection among IDUs in Iran is 50%-75% [9]. Similar results were reported in some previous studies [1–3, 6], while others have shown higher prevalence among older age groups [4, 21, 35]. This may be due to some differences in social life styles, level of awareness, predominant routes of transmission, and risk behavior patterns among different age groups in different parts of the world.

The prevalence of HIV infection among blood donors in our study was reported to be 0.004%, which is lower than those of Mangalore (0.1%) [1], Ethiopia (3.8%) [25], China (0.31%) [21], Nepal (0.21%) [2], and Nigeria (1.4%) [6] but higher than those of Australia (0.0003%) [28], Italy (0.00019%) [29], and Mongolia (0%) [26]. These variations in the prevalence of HIV might be due to some differences in risk behaviors, educational programs, preventive measures, and quality of safety measures employed in blood transfusion centers of those countries. The reported prevalence in this study is also in line with the previous reports from Iran, 0.0054% [19], 0.004% [8, 18].

The seroprevalence of all screened viral infections in this study was lower among female donors than in male donors. Previous studies have also reported the similar observation in many countries [2, 3, 8, 19, 34, 37]. This might be due to lesser contribution of women in social activities and high-risk behaviors such as multiple sex relationships and intravenous drug use. Therefore, these gender differences in infection rates reflect some differences in lifestyles, social activities, and sexual behavior between these two genders.

The results of the present study in accordance with those of other studies indicate that the prevalence of viral infections is higher among first-time donors compared to regular and repeated donors, while regular donors have the lowest frequency of these three pathogens [1, 8, 19, 24, 25]. The reason may be that the regular donors are well-informed individuals with low risk behaviors due to several time screening and selection in the process of donating blood [1, 5, 19, 21, 25]. Therefore, regular donors should be encouraged to participate more in blood donation to further improve the safety of blood and reduce the risk of TTIs in the society. Moreover, in line with the previous studies [19, 24], higher rates of the viral infections were found among low educated donors in the present study. Better knowledge of highly educated donors regarding TTIs, their routes of transmission and risk factors can explain this finding. On the other hand, it is worthy to note that frequently people with lower level of education live in worse conditions, which increase their vulnerability to infections. Therefore, nationwide efforts should be made to raise public awareness in people with lower level of education to reduce the risk of transmission.

This study clearly indicates declining trends in the seroprevalence of HBV and HCV in donated blood from 2004 to 2014, while HIV had a slight but not significant decline. Similar decreasing trends have also been reported in previous studies from Iran [8, 17, 18, 20, 24] as well as some other countries such as Argentina [13], Pakistan [22], Ethiopia [25], Saudi Arabia [30], Lebanon [38], and Canada [27]. These declining trends might be a reflection of a decline in the rates of these infections in the society. However, some other factors, including vaccination against HBV infection, an increase in public awareness regarding general prevalence and transmission routes of these viral infections, progress in selection of a safer donor population through application of more standard questionnaire and more effective procedures in physical examination by trained medical doctors, application of confidential unit exclusion, which allows self-deferral of high risk donors prior to donation, automation of data registry of all donors, systematic screening of all donations for infectious markers, improvement in donor screening procedures through application of more sensitive screening kits, improvement in safety measures through application of standard instruments and operating procedures, validation of all procedures across the country, an increase in the number of regular blood donors from 1716 (14.9%) in 2004 to 19822 (56.6%) in 2014, educational program regarding blood donation to improve the blood safety, and progress in preventive measures might also explain such declines in our study.

Despite having common routes of transmission and similar risk factors [6, 35], the prevalence of HBV was higher than HCV and HIV in the present study. The similar finding has also been reported in previous studies from Iran [8, 18, 19]. The reason of this high prevalence may be higher infectivity of HBV compared to HCV and HIV [34, 39]. Previous studies have also confirmed this issue [20, 34, 39].

Despite indicating the time trends of these viral infections, a fair comparison between the years analyzed is not possible, since many factors such as donor selection criteria, sensitivity and specificity of screening kits used, level of awareness, population risks, behavioral factors, prevention programs, and the safety measures employed in blood transfusion centers may change over the years. This is one of the limitations of the current study. Nevertheless, all of these changes have resulted in a considerable decline in the prevalence of HBV and HCV over the years.

## Conclusion

The results of the present study confirm the effectiveness of donor screening and selecting policy employed by IBTO in recent years. Despite these improvements, still a long way is ahead to achieve a zero-risk blood transfusion. Majority of risks are due to blood donation during the serologically negative window period or asymptomatic phase of infection as well as the possible presence of apparently healthy donors with occult infections. During these intervals, blood transfusion is capable of transmitting infection despite negative serological screening tests [1, 9, 30, 39]. Undetectable transmission of viral infections poses a serious threat to blood safety [1, 25]. Fortunately, the most of these unnoticeable transmissions are preventable through application of nucleic acid-based detection techniques such as polymerase chain reaction (PCR) [21, 30, 37].

The introduction of nucleic acid amplification techniques (NAT) in the realm of diagnosis of viral infections caused a great revolution in the transfusion medicine. However, PCR is quite successful in minimizing the threat of unnoticeable infection [37, 40] but is financially and technically beyond reach of the Iranian blood bank transfusion centers. Currently, routine screening of blood donations relies on serological tests in Iran [9, 17]. Therefore, it is important for IBTO to focus on proper selection of donors, continuous screening of donated blood for infectious markers, and improvement of preventive strategies as well as public awareness to improve the safety of the blood transfusion in Iran. In addition, rational transfusion of blood only when necessity arises is also essential to reduce the risk of transmission of these infections through blood transfusion.
